# Implementable tensor methods in unconstrained convex optimization

**DOI:** 10.1007/s10107-019-01449-1

**Published:** 2019-11-21

**Authors:** Yurii Nesterov

**Affiliations:** Center for Operations Research and Econometrics (CORE), Catholic University of Louvain (UCL), Louvain-la-Neuve, Belgium

**Keywords:** High-order methods, Tensor methods, Convex optimization, Worst-case complexity bounds, Lower complexity bounds, 90C25, 90C06, 65K05

## Abstract

In this paper we develop new tensor methods for unconstrained convex optimization, which solve at each iteration an auxiliary problem of minimizing convex multivariate polynomial. We analyze the simplest scheme, based on minimization of a regularized local model of the objective function, and its accelerated version obtained in the framework of estimating sequences. Their rates of convergence are compared with the worst-case lower complexity bounds for corresponding problem classes. Finally, for the third-order methods, we suggest an efficient technique for solving the auxiliary problem, which is based on the recently developed relative smoothness condition (Bauschke et al. in Math Oper Res 42:330–348, 2017; Lu et al. in SIOPT 28(1):333–354, 2018). With this elaboration, the third-order methods become implementable and very fast. The rate of convergence in terms of the function value for the accelerated third-order scheme reaches the level $O\left( {1 \over k^4}\right)$, where *k* is the number of iterations. This is very close to the lower bound of the order $O\left( {1 \over k^5}\right)$, which is also justified in this paper. At the same time, in many important cases the computational cost of one iteration of this method remains on the level typical for the second-order methods.

## Introduction

*Motivation* In the last decade, we observe an increasing interest to the complexity analysis of the high-order methods. Starting from the paper [31] containing the first global rate of convergence of Cubic Regularization of Newton Method, it became more and more common to provide the second-order methods with the worst-case complexity bounds on different problem classes (see, for example, [5, 11, 12]). New efficiency measurements in this field naturally generated a new spectrum of questions, starting from the possibilities to accelerate the second-order methods (see [27]) up to the lower complexity bounds (see [1, 2, 13, 18]) and attempts of constructing the optimal methods [24].

Another possibility of accelerating the minimization processes consists in the increase of the power of oracle. The idea of using the high-order approximations in Optimization is not new. Initially, such approximations were employed in the optimality conditions (see, for example [22]). However, it seems that the majority of attempts of using the high-order tensors in optimization methods failed by the standard obstacle related to the enormous complexity of minimization of nonconvex multivariate polynomials. To the best of our knowledge, the only theoretical analysis of such schemes for convex problems can be found in an unpublished preprint [3], which is concluded by a pessimistic comment on practical applicability of these methods. For nonconvex problems, several recent papers [8–10, 14] contain the complexity analysis for high-order methods designed for generating points with small norm of the gradient. For the auxiliary nonconvex optimization problem, these methods need to guarantee a sufficient level for the first-order optimality condition and the local decrease of the objective function. However, for nonconvex functions even this moderate goal is difficult to achieve.

The key observation, which underlies all results of this paper, is that an appropriately regularized Taylor approximation of convex function is a convex multivariate polynomial. This is indeed a very natural property since this regularized approximation usually belongs to the epigraph of convex function. Thus, the auxiliary optimization problem in the high-order (or *tensor*) methods becomes generally solvable by many powerful methods of Convex Optimization. This fact explains our interest to complexity analysis of the simplest tensor scheme (Sect. 2), based on the *convex* regularized Taylor approximation, and to its accelerated version (Sect. 3). The latter method is obtained by the technique of estimating functions (see [25–27]). Therefore it is similar to Algorithm 4.2 in [3]. The main difference consists in the correct choice of parameters ensuring convexity of the auxiliary problem. We show that this algorithm converges with the rate $O(\left( {1 \over k}\right) ^{p+1})$, where *k* is the number of iterations and *p* is the degree of the tensor.

In the next Sect. 4, we derive lower complexity bounds for the tensor methods. We show that the lower bound for the rate of convergence is of the order $O\left( \left( {1 \over k} \right) ^{3p+1 \over 2}\right)$. This result is better than the bound in [1] and coincide with the bound in [2]. However, it seems that our justification is simpler.

For practical implementations, the most important results are included in Sect. 5, where we discuss an efficient scheme for minimizing the regularized Taylor approximation of degree three. This auxiliary convex problem can be treated in the framework of *relative smoothness condition*. The first element of this approach was introduced in [4], for generalizing the Lipschitz condition for the norm of the gradient. In [21] it was shown that the same extension can be applied to the condition of strong convexity. This second step is important since it leads to linearly convergent methods for functions with nonstandard growth properties. The auxiliary problem with the third-order tensor is a good application of this technique. We show that the corresponding method converges linearly, with the rate depending on an absolute constant. In the end of the section, we argue that the complexity of one iteration of the resulting third-order scheme is often of the same order as that of the second-order methods.

In the last Sect. 6 we discuss the presented results and mention the open problems.

*Notations and generalities* In what follows, we denote by $\mathbb {E}$ a finite-dimensional real vector space, and by $\mathbb {E}^*$ its dual spaced composed by linear functions on $\mathbb {E}$. For such a function $s \in \mathbb {E}^*$, we denote by $\langle s, x \rangle$ its value at $x \in \mathbb {E}$. Using a self-adjoint positive-definite operator $B: \mathbb {E}\rightarrow \mathbb {E}^*$ (notation $B = B^* \succ 0$), we can endow these spaces with *conjugate Euclidean norms*:

$$\begin{aligned} \begin{array}{rcl} \Vert x \Vert= & {} \langle B x, x \rangle ^{1/2}, \quad x \in \mathbb {E}, \quad \Vert g \Vert _* \; = \; \langle g, B^{-1} g \rangle ^{1/2}, \quad g \in E^*. \end{array} \end{aligned}$$

Sometimes, in the formulas involving products of linear operators, it will be convenient to treat $x \in \mathbb {E}$ as a linear operator from ${\mathbb {R}}$ to $\mathbb {E}$, and $x^*$ as a linear operator from $\mathbb {E}^*$ to ${\mathbb {R}}$. In this case, $xx^*$ is a linear operator from $\mathbb {E}^*$ to $\mathbb {E}$, acting as follows:

$$\begin{aligned} \begin{array}{rcl} (xx^*)g= & {} \langle g, x \rangle x \; \in \; \mathbb {E}, \quad g \in \mathbb {E}^*. \end{array} \end{aligned}$$

For a smooth function $f: {\mathrm{dom }}\,f \rightarrow {\mathbb {R}}$ with convex and open domain ${\mathrm{dom }}\,f \subseteq \mathbb {E}$, denote by $\nabla f(x)$ its gradient, and by $\nabla ^2 f(x)$ its Hessian evaluated at point $x \in {\mathrm{dom }}\,f \subseteq \mathbb {E}$. Note that

$$\begin{aligned} \begin{array}{rcl} \nabla f(x)\in & {} \mathbb {E}^*, \quad \nabla ^2 f(x) h \; \in \; \mathbb {E}^*, \quad x \in {\mathrm{dom }}\,f, \; h \in \mathbb {E}. \end{array} \end{aligned}$$

In what follows, we often work with directional derivatives. For $p \ge 1$, denote by

$$\begin{aligned} \begin{array}{c} D^p f(x)[h_1, \dots , h_p] \end{array} \end{aligned}$$

the directional derivative of function *f* at *x* along directions $h_i \in \mathbb {E}$, $i = 1, \dots , p$. Note that $D^p f(x)[ \cdot ]$ is a *symmetric* *p-linear form*. Its *norm* is defined in the standard way:

1.1 $$\begin{aligned} \begin{array}{rcl} \Vert D^pf(x) \Vert= & {} \max \limits _{h_1, \dots , h_p} \left\{ D^p f(x)[h_1, \dots , h_p ]: \; \Vert h_i \Vert \le 1, \, i = 1, \dots , p \right\} . \end{array} \end{aligned}$$

For example, for any $x \in {\mathrm{dom }}\,f$ and $h_1, h_2 \in \mathbb {E}$, we have

$$\begin{aligned} \begin{array}{rcl} Df(x)[h_1]= & {} \langle \nabla f(x), h_1 \rangle , \quad D^2f(x)[h_1, h_2] \; = \; \langle \nabla ^2 f(x) h_1, h_2 \rangle . \end{array} \end{aligned}$$

Thus, for the Hessian, our definition corresponds to the *spectral norm* of self-adjoint linear operator (maximal module of all eigenvalues computed with respect to operator *B*).

If all directions $h_1, \dots , h_p$ are the same, we apply notation

$$\begin{aligned} \begin{array}{c} D^p f(x)[h]^p, \quad h \in \mathbb {E}. \end{array} \end{aligned}$$

Then, Taylor approximation of function $f(\cdot )$ at $x \in {\mathrm{dom }}\,f$ can be written as follows:

$$\begin{aligned} \begin{array}{rcl} f(x+h) &{} = &{} \Phi _{x,p}(h) + o(\Vert h\Vert ^p), \quad x+h \in {\mathrm{dom }}\,f,\\ \\ \Phi _{x,p}(y) &{} {\mathop {=}\limits ^{{\mathrm {def}}}}&{} f(x) + \sum \limits _{i=1}^p {1 \over i!} D^if(x)[y-x]^i, \quad y \in \mathbb {E}. \end{array} \end{aligned}$$

Note that in general, we have (see, for example, Appendix 1 in [30])

1.2 $$\begin{aligned} \begin{array}{rcl} \Vert D^pf(x) \Vert= & {} \max \limits _{h} \left\{ \Big | D^p f(x)[h]^p \Big |: \; \Vert h \Vert \le 1 \right\} . \end{array} \end{aligned}$$

Similarly, since for $x, y \in {\mathrm{dom }}\,f$ being fixed, the form $D^pf(x)[\cdot , \dots , \cdot ] - D^pf(y)[\cdot , \dots , \cdot ]$ is *p*-linear and symmetric, we also have

1.3 $$\begin{aligned} \Vert D^pf(x) - D^pf(y) \Vert= & {} \max \limits _{h} \left\{ \Big | D^p f(x)[h]^p - D^pf(y)[h]^p\Big |: \; \Vert h \Vert \le 1 \right\} .\nonumber \\ \end{aligned}$$

In this paper, we consider functions from the problem classes $\mathcal{F}_p$, which are convex and *p* times differentiable on $\mathbb {E}$. Denote by $L_p$ its uniform bound for the Lipschitz constant of their *p*th derivative:

1.4 $$\begin{aligned} \begin{array}{rcl} \Vert D^p f(x) - D^pf(y) \Vert\le & {} L_p \Vert x - y \Vert , \quad x, y \in {\mathrm{dom }}\,f, \quad p \ge 1. \end{array} \end{aligned}$$

Sometimes, if an ambiguity can arise, we us notation $L_p(f)$.

Assuming that $f \in \mathcal{F}_{p}$ and $L_{p} < +\infty$, by the standard integration arguments we can bound the residual between function value and its Taylor approximation:

1.5 $$\begin{aligned} \begin{array}{rcl} | f(y) - \Phi _{x,p}(y) |\le & {} {L_{p} \over (p+1)!} \Vert y - x \Vert ^{p+1}, \quad x, y \in {\mathrm{dom }}\,f. \end{array} \end{aligned}$$

If $p \ge 2$, then applying the same reasoning for functions $\langle \nabla f(\cdot ), h \rangle$ and $\langle \nabla ^2 f(\cdot ) h, h \rangle$ with direction $h \in \mathbb {E}$ being fixed, we get the following guarantees:

1.6 $$\begin{aligned}&\begin{array}{rcl} \Vert \nabla f(y) - \nabla \Phi _{x,p}(y) \Vert _*\le & {} {L_p \over p!} \Vert y - x \Vert ^{p}, \end{array} \end{aligned}$$

1.7 $$\begin{aligned}&\begin{array}{rcl} \Vert \nabla ^2 f(y) - \nabla ^2 \Phi _{x,p}(y) \Vert\le & {} {L_p \over (p-1)!} \Vert y - x \Vert ^{p-1}, \end{array} \end{aligned}$$

which are valid for all $x, y \in {\mathrm{dom }}\,f$.

## Convex tensor approximations

In our methods, we use the following *power prox function*

2.1 $$\begin{aligned} \begin{array}{rcl} d_p(x)= & {} {1 \over p} \Vert x \Vert ^p, \quad p \ge 2. \end{array} \end{aligned}$$

Note that

2.2 $$\begin{aligned} \begin{array}{rcl} \nabla d_p(x) &{} = &{} \Vert x \Vert ^{p-2} Bx, \\ \\ \nabla ^2 d_p(x) &{} = &{} (p-2)\Vert x \Vert ^{p-4} Bx x^* B + \Vert x \Vert ^{p-2} B \\ \\ &{} \succeq &{} \Vert x \Vert ^{p-2} B. \end{array} \end{aligned}$$

All results of this paper are based on the following observation. From now on, for the sake of simplicity, we assume that ${\mathrm{dom }}\,f = \mathbb {E}$.

### Theorem 1

Let $f \in \mathcal{F}_p$ with $p \ge 2$ and $L_p < +\infty$. Then for any $x, y \in \mathbb {E}$ we have

2.3 $$\begin{aligned} \begin{array}{rcl} \nabla ^2 f(y)\preceq & {} \nabla ^2 \Phi _{x,p}(y) + {L_p \over (p-1)!} \Vert y - x \Vert ^{p-1} B. \end{array} \end{aligned}$$

Moreover, for any $M \ge L_p$ and any $x \in \mathbb {E}$, function^1^

2.4 $$\begin{aligned} \begin{array}{rcl} \Omega _{x,p,M}(y)= & {} \Phi _{x,p}(y) + {M \over (p-1)!} d_{p+1}(y - x) \end{array} \end{aligned}$$

is convex and

2.5 $$\begin{aligned} \begin{array}{rcl} f(y)\le & {} \Omega _{x,p,M}(y), \quad y \in \mathbb {E}. \end{array} \end{aligned}$$

### Proof

Let us fix arbitrary *x* and *y* from ${\mathrm{dom }}\,f$. Then for any direction $h \in \mathbb {E}$ we have

$$\begin{aligned} \begin{array}{rcl} \langle (\nabla ^2 f(y) - \nabla ^2 \Phi _{x,p} (y))h,h \rangle &{} \le &{} \Vert \nabla ^2 f(y) - \nabla ^2 \Phi _{x,p} (y) \Vert \cdot \Vert h \Vert ^2\\ \\ &{} {\mathop {\le }\limits ^{({\mathrm{1.7}})}} &{} {L_p \over (p-1)!} \Vert y - x \Vert ^{p-1} \Vert h \Vert ^2, \end{array} \end{aligned}$$

and this is (2.3). Further,

$$\begin{aligned} \begin{array}{rcl} 0 \; \preceq \; \nabla ^2 f(y) &{} {\mathop {\preceq }\limits ^{({\mathrm{2.3}})}} &{} \nabla ^2 \Phi _{x,p}(y) + {L_p \over (p-1)!} \Vert y - x \Vert ^{p-1} B\\ \\ &{} \preceq &{} \nabla ^2 \Phi _{x,p}(y) + {M \over (p-1)!} \Vert y - x \Vert ^{p-1} B \\ \\ &{} {\mathop {\preceq }\limits ^{({\mathrm{2.2}})}} &{} \nabla ^2 \Phi _{x,p}(y) + {M \over (p-1)!} \nabla ^2 d_{p+1}(y-x)\\ \\ &{} {\mathop {=}\limits ^{({\mathrm{2.4}})}} &{} \nabla ^2 \Omega _{x,p,M}(y). \end{array} \end{aligned}$$

Thus, function $\Omega _{x,p,M}(\cdot )$ is convex. Finally,

$$\begin{aligned} \begin{array}{rcl} f(y) &{} {\mathop {\le }\limits ^{({\mathrm{1.5}})}} &{} \Phi _{x,p}(y) + {L_p \over (p+1)!} (p+1) d_{p+1}(y-x) \\ \\ &{} \le &{} \Phi _{x,p}(y) + {M \over p!} d_{p+1}(y-x)\\ \\ &{} \le &{} \Omega _{x,p,M}(y). \end{array} \end{aligned}$$

$$\square $$

The statements of Theorem 1 explain our interest to the following point:

2.6 $$\begin{aligned} \begin{array}{rcl} T_{p,M}(x)\in & {} \text{ Arg }\min \limits _{y \in \mathbb {E}} \Omega _{x,p,M}(y) \end{array} \end{aligned}$$

with $M \ge L_p$. We are going to use such points for solving the problem

2.7 $$\begin{aligned} \begin{array}{rcl} f_*= & {} \min \limits _{x \in \mathbb {E}} f(x), \end{array} \end{aligned}$$

starting from some point $x_0 \in \mathbb {E}$, where $f \in \mathcal{F}_p$ and $L_p < +\infty$. We assume that there exists at least one solution $x_*$ of this problem and that the level sets of *f* are bounded:

2.8 $$\begin{aligned}&\max \limits _{x \in \mathcal{L}(x_0)} \Vert x - x_* \Vert \; \le \; D \; < \; + \infty ,\nonumber \\&\mathcal{L}(x_0) \; {\mathop {=}\limits ^{{\mathrm {def}}}}\; \{ x \in \mathbb {E}: \; f(x) \le f(x_0) \}. \end{aligned}$$

In this case, in view of (2.5), the level sets of function $\Omega _{x,p,M}(\cdot )$ are also bounded. Therefore, point $T = T_{p,M}(x)$ is well defined. It satisfies the following first-order optimality condition:

2.9 $$\begin{aligned} \begin{array}{rcl} \nabla \Phi _{x,p}(T) + {M \over (p-1)!} \Vert T - x \Vert ^{p-1} B(T-x)= & {} 0. \end{array} \end{aligned}$$

Multiplying this equality by $T-x$, we get

2.10 $$\begin{aligned} \begin{array}{rcl} {M \over (p-1)!} \Vert T - x \Vert ^{p+1}= & {} \langle \nabla \Phi _{x,p}(T), x - T \rangle . \end{array} \end{aligned}$$

Denote $$r_{p,M}(x) = \Vert x - T_{p,M}(x) \Vert $$.

### Lemma 1

For any $x \in \mathbb {E}$ and $M \ge L_p$ we have

2.11 $$\begin{aligned}&\begin{array}{rcl} f(T_{p,M}(x))\le & {} \min \limits _{y \in \mathbb {E}} \left\{ f(y) + {p M + L_p \over (p+1)!} \Vert y - x \Vert ^{p+1} \right\} , \end{array} \end{aligned}$$

2.12 $$\begin{aligned}&\begin{array}{rcl} \Vert \nabla f(T_{p,M}(x)) \Vert _*\le & {} {pM+L_p \over p!} \Vert x - T_{p,M}(x) \Vert ^{p}, \end{array} \end{aligned}$$

2.13 $$\begin{aligned}&\begin{array}{rcl} \langle \nabla f(T_{p,M}(x)), x - T_{p,M}(x) \rangle\ge & {} {(p-1)! \over 2 M r_{p,M}^{p-1}(x)} \Vert \nabla f(T_{p,M}(x)) \Vert _*^2 + {M^2 - L^2_p \over 2M (p-1)!} r_{p,M}^{p+1}(x). \end{array}\nonumber \\ \end{aligned}$$

First two inequalities of this lemma are already known (see, for example, [8]). We provide them with a simple proof for the reader convenience.

### Proof

Denote $T = T_{p,M}(x)$ and $r = \Vert x - T \Vert$. Then,

$$\begin{aligned} \begin{array}{rcl} f(T) &{} {\mathop {\le }\limits ^{({\mathrm{2.5}})}} &{} \min \limits _{y \in \mathbb {E}} \Omega _{x,p,M}(y) \; = \; \min \limits _{y \in \mathbb {E}} \left\{ \Phi _{p,x}(y) + {pM \over (p+1)!} \Vert y - x \Vert ^{p+1} \right\} \\ \\ &{} {\mathop {\le }\limits ^{({\mathrm{1.5}})}} &{} \min \limits _{y \in \mathbb {E}} \left\{ f(y) + {pM + L_p \over (p+1)!} \Vert y - x \Vert ^{p+1} \right\} . \end{array} \end{aligned}$$

and this is inequality (2.11). Further,

2.14 $$\begin{aligned} \begin{array}{rcl} \Vert \nabla f(T) - \nabla \Phi _{x,p}(T) \Vert _*&{\mathop {\le }\limits ^{({\mathrm{1.6}})}}&{L_p \over p!} r^p. \end{array} \end{aligned}$$

Therefore, we get the following bound:

$$\begin{aligned} \begin{array}{rcl} \Vert \nabla f(T) \Vert _* &{} \le &{} \Vert \nabla f(T) - \nabla \Phi _{x,p}(T) \Vert _* + \Vert \nabla \Phi _{x,p}(T)\Vert _*\\ \\ &{} {\mathop {\le }\limits ^{({\mathrm{2.14}})}} &{} {L_p \over p!} r^p + \Vert \nabla \Phi _{x,p}(T)\Vert _* \\ \\ &{} {\mathop {=}\limits ^{({\mathrm{2.9}})}} &{} \left( {L_p \over p!} +{M \over (p-1)!} \right) r^p, \end{array} \end{aligned}$$

which leads to (2.12). Finally,

$$\begin{aligned} \begin{array}{rcl} \Vert \nabla f(T) + {M r^{p-1} \over (p-1)!} B (T - x ) \Vert _*&{\mathop {=}\limits ^{({\mathrm{2.9}})}}&\Vert \nabla f(T) - \nabla \Phi _{x,p}(T) \Vert _* \; {\mathop {\le }\limits ^{({\mathrm{2.14}})}} \; {L_p \over (p-1)!} r^p. \end{array} \end{aligned}$$

Squaring both sides of this bound, we get:

$$\begin{aligned} \begin{array}{rcl} \Vert \nabla f(T) \Vert ^2_* + {2 M r^{p-1} \over (p-1)!} \langle \nabla f(T), T-x \rangle + {M^2 r^{2p} \over [(p-1)!]^2}\le & {} {L^2_{p+1} r^{2p}\over [(p-1)!]^2}, \end{array} \end{aligned}$$

and this is (2.13). $\square$

### Corollary 1

For any $x \in \mathbb {E}$ and $M \ge L_p$, we have

2.15 $$\begin{aligned} \begin{array}{rcl} \langle \nabla f(T_{p,M}(x), x - T_{p,M}(x) \rangle\ge & {} {c(p) \over M} [M^2 - L_p^2]^{p-1 \over 2p} \Vert \nabla f(T_{p,M}(x)) \Vert ^{p+1 \over p}_*, \end{array} \end{aligned}$$

where $$c(p) = {p \over p-1} \left[ {p-1 \over p+1} \right] ^{1-p \over 2p} [(p-1)!]^{1 \over p}$$.

### Proof

Indeed, in view of inequality (2.13), we have the following bound:

$$\begin{aligned} \begin{array}{rcl} \langle \nabla f(T), x - T \rangle\ge & {} {a \over \tau } + b \tau ^{\alpha }, \end{array} \end{aligned}$$

where $a = {(p-1)! \over 2 M} \Vert \nabla f(T_{p,M}(x)) \Vert _*^2$, $b = {M^2 - L^2_p \over 2M (p-1)!}$, $\tau = r_{p,M}^{p-1}(x)$, and $\alpha = {p+1 \over p-1}$. Note that

$$\begin{aligned} \begin{array}{rcl} \min \limits _{\tau > 0} \left\{ {a \over \tau } + b \tau ^{\alpha } \right\}= & {} (1+\alpha ) \left( a \over \alpha \right) ^{\alpha \over 1+\alpha } b^{1 \over 1 + \alpha }. \end{array} \end{aligned}$$

It remains to substitute in this bound the values of our parameters *a*, *b*, and $\alpha$. $\square$

Let us estimate now the rate of convergence of the following process:2.16where $M \ge L_p$. Thus, in view of Theorem 1, point $x_{t+1}$ is a solution to the auxiliary convex problem (2.6).

### Theorem 2

Let sequence $\{ x_t \}_{t \ge 0}$ be generated by method (2.16) as applied to problem (2.7). Then for all $t \ge 0$ we have $f(x_{t+1}) \le f(x_t)$. At the same time,

2.17 $$\begin{aligned} \begin{array}{rcl} f(x_{t}) - f_*\le & {} {(pM + L_p) D^{p+1} \over (p+1)! \left( 1 + (t-1)\left( {1 \over p+1}\right) ^{{p+1 \over p}} \right) ^{p} } \; \le \; {(pM + L_p) D^{p+1} \over p!} \left( {p + 1\over t}\right) ^p, \quad t \ge 1. \end{array}\nonumber \\ \end{aligned}$$

### Proof

In view of inequality (2.5), method (2.16) is monotone. Hence, for all $t \ge 0$ we have

2.18 $$\begin{aligned} \begin{array}{rcl} \Vert x_t - x_* \Vert\le & {} D. \end{array} \end{aligned}$$

Let us prove the first inequality in (2.17). First of all, let us estimate the difference $f(x_1) - f_*$. We have

$$\begin{aligned} \begin{array}{rcl} f(x_1)&{\mathop {\le }\limits ^{({\mathrm{2.11}})}}&\min \limits _{y \in \mathbb {E}} \left\{ f(y) + {p M + L_p \over (p+1)!} \Vert y - x_0 \Vert ^{p+1} \right\} \; {\mathop {\le }\limits ^{({\mathrm{2.18}})}} \; f_* + {(pM + L_p) D^{p+1} \over (p+1)!}, \end{array} \end{aligned}$$

and this is (2.17) for $t = 1$.

Further, for any $t \ge 1$, we have

$$\begin{aligned} \begin{array}{rcl} f(x_{t+1}) &{} {\mathop {\le }\limits ^{({\mathrm{2.11}})}} &{} \min \limits _{y \in \mathbb {E}} \left\{ f(y) + {p M + L_p \over (p+1)!} \Vert y - x_t \Vert ^{p+1} \right\} \\ \\ &{} {\mathop {\le }\limits ^{({\mathrm{2.18}})}} &{} \min \limits _{\alpha \in [0,1]} \left\{ f(x_t + \alpha (x_*-x_t)) + {(p M + L_p)D^{p+1} \over (p+1)!} \alpha ^{p+1} \right\} \\ \\ &{} \le &{} \min \limits _{\alpha \in [0,1]} \left\{ f(x_t) - \alpha (f(x_t)-f_*) + {(p M + L_p)D^{p+1} \over (p+1)!} \alpha ^{p+1} \right\} . \end{array} \end{aligned}$$

The minimum of the above objective in $\alpha \ge 0$ is achieved for

$$\begin{aligned} \begin{array}{rcl} \alpha _*= & {} \left( {(f(x_t)-f_*) p! \over (p M + L_p)D^{p+1} } \right) ^{1 \over p} \; \le \; \left( {(f(x_1)-f_*) p! \over (p M + L_p)D^{p+1} } \right) ^{1 \over p} \; {\mathop {\le }\limits ^{({\mathrm{2.17}})}} \; \left( {1 \over p+1} \right) ^{1 \over p} \; < \; 1. \end{array} \end{aligned}$$

Thus, we conclude that

$$\begin{aligned} \begin{array}{rcl} f(x_{t+1}) &{} \le &{} f(x_t) - \alpha _* \left( f(x_t)-f_* - {(p M + L_p)D^{p+1} \over (p+1)!} \alpha _*^{p} \right) \\ \\ &{} = &{} f(x_t) - {p \, \alpha _* \over p+1} (f(x_t)-f_*). \end{array} \end{aligned}$$

Denoting $\delta _t = f(x_t) - f_*$, we get the following estimate:

$$\begin{aligned} \delta _t - \delta _{t+1} \ge C \delta _t ^{p+1 \over p}, \quad t \ge 1, \end{aligned}$$

where $C = {p \over p+1}\left( {p! \over (p M + L_p)D^{p+1} } \right) ^{1 \over p}$. Thus, for $\mu _t = C^p \delta _t$, the recursive inequality is as follows:

$$\begin{aligned} \mu _t - \mu _{t+1} \ge \mu ^{p+1 \over p}_t, \quad t \ge 1. \end{aligned}$$

Then,

$$\begin{aligned} \begin{array}{rcl} {1 \over \mu _{t+1}^{1/p}} - {1 \over \mu _{t}^{1/p}} &{} = &{} {\mu _{t}^{1/p} - \mu _{t+1}^{1/p} \over \mu _{t+1}^{1/p}\mu _{t}^{1/p}} \; = \; {1 \over \mu _{t+1}^{1/p}\mu _{t}^{1/p}} \left( \mu _{t}^{1/p} - \mu _t^{1/p} \left( 1 +{\mu _{t+1}- \mu _t \over \mu _t} \right) ^{1/p} \right) \\ \\ &{} \ge &{} {1 \over \mu _{t+1}^{1/p}\mu _{t}^{1/p}} \left( \mu _{t}^{1/p} - \mu _t^{1/p} \left( 1 +{\mu _{t+1}- \mu _t \over p \mu _t} \right) \right) \; = \; {\mu _{t}- \mu _{t+1} \over p \mu _t \mu _{t+1}^{1/p}} \\ \\ &{} \ge &{} {\mu _{t}^{1/p} \over p \mu _{t+1}^{1/p}} \; \ge \; {1 \over p}. \end{array} \end{aligned}$$

This means that ${1 \over \mu _t} \ge \left( {1 \over \mu _1^{1/p}} + {t-1 \over p} \right) ^p$. Note that

$$\begin{aligned} \begin{array}{rcl} {1 \over \mu _1^{1/p}}= & {} {1 \over C \delta _1^{1/p}} \; = \; {p+1 \over p} \left( {(pM + L_p) D^{p+1} \over p! (f(x_1) - f_*) } \right) ^{1 \over p} \; {\mathop {\ge }\limits ^{({\mathrm{2.17}})}} \; {1 \over p} (p+1)^{p+1 \over p}. \end{array} \end{aligned}$$

Therefore,

$$\begin{aligned} \begin{array}{rcl} \delta _t &{} = &{} C^{-p} \mu _t \; = \; \left( {p+1 \over p}\right) ^p {(pM + L_p) D^{p+1} \over p!} \mu _t\\ \\ &{} \le &{} \left( {p+1 \over p}\right) ^p {(pM + L_p) D^{p+1} \over p!} \left( {1 \over p} (p+1)^{p+1 \over p} + {t-1 \over p} \right) ^{-p}\\ \\ &{} = &{} {(pM + L_p) D^{p+1} \over p!} \left( (p+1)^{1/p} + {t-1 \over p+1} \right) ^{-p}, \end{array} \end{aligned}$$

and this is (2.17). $\square$

## Accelerated tensor methods

In order to accelerate method (2.16), we apply a variant of the *estimating sequences technique*, which becomes a standard tool for accelerating the usual Gradient and Second-Order Methods (see, for example, [25–27]). In our situation, this idea can be applied to tensor methods in the following way.

For solving the problem (2.7), we choose a constant $M \ge L_p$ and recursively update the following sequences.

- Sequence of estimating functions
  3.1 $$\begin{aligned} \begin{array}{rcl} \psi _k(x)= & {} \ell _k(x) + {C \over p!} d_{p+1}(x-x_0), \quad k = 1, 2, \dots , \end{array} \end{aligned}$$
  where $\ell _k(x)$ are linear functions in $x \in \mathbb {E}$, and *C* is a positive parameter.
- Minimizing sequence $$\{ x_k \}_{k=1}^{\infty }$$.
- Sequence of scaling parameters $\{A_k \}_{k=1}^{\infty }$:
  $$\begin{aligned} A_{k+1} \; {\mathop {=}\limits ^{{\mathrm {def}}}}\; A_k + a_k, \quad k = 1,2, \dots . \end{aligned}$$

For these objects, we are going to maintain the following relations:

3.2 $$\begin{aligned} \left. \begin{array}{l} \mathcal{R}^1_k: A_k f(x_{k}) \le \psi _k^* \; \equiv \; \min \limits _{x \in \mathbb {E}} \psi _k(x),\\ \\ \mathcal{R}^2_k: \psi _k(x) \le A_k f(x) + {p M + L_p + C \over p!} d_{p+1}(x - x_0),\; \forall x \in \mathbb {E}. \end{array} \right\} , \quad k \ge 1. \end{aligned}$$

Let us ensure that relations (3.2) hold for $k = 1$. We choose

3.3 $$\begin{aligned} \begin{array}{c} x_1 \; = \; T_{p,M}(x_0),\quad \ell _1(x) \; \equiv \; f(x_1),\; x \in \mathbb {E}, \quad A_1 \; = \;1. \end{array} \end{aligned}$$

Then $\psi _1^* = f(x_1)$, so $\mathcal{R}_1^1$ holds. On the other hand, in view of definition (3.1), we get

$$\begin{aligned} \begin{array}{rcl} \psi _1(x) &{} = &{} f(x_1) + {C \over p!} d_{p+1}(x - x_0) \\ \\ &{} {\mathop {\le }\limits ^{({\mathrm{2.11}})}} &{} \min \limits _{y \in \mathbb {E}} \left[ f(y) + {p M + L_p \over (p+1)!} \Vert y - x_0 \Vert ^{p+1} \right] + {C \over p!}d_{p+1}( x - x_0), \end{array} \end{aligned}$$

and $$\mathcal{R}_1^2$$ follows.

Assume now that relations (3.2) hold for some $k \ge 1$. Denote

$$\begin{aligned} v_k \; = \; \arg \min \limits _{x \in \mathbb {E}} \; \psi _k(x). \end{aligned}$$

Let us choose some $a_k > 0$ and $M \ge L_p$. Define

3.4 $$\begin{aligned} \begin{array}{rcl} \alpha _k &{} = &{} {a_k \over A_k + a_k}, \quad y_k \; = \; (1 -\alpha _k) x_k + \alpha _k v_k, \quad x_{k+1} \; = \; T_{p,M}(y_k),\\ \\ \psi _{k+1}(x) &{} = &{} \psi _k(x) + a_k [f(x_{k+1}) + \langle \nabla f(x_{k+1}), x - x_{k+1} \rangle ]. \end{array} \end{aligned}$$

In view of $\mathcal{R}_k^2$ and convexity of *f*, for any $x \in \mathbb {E}$ we have

$$\begin{aligned} \psi _{k+1}(x)\le & {} A_k f(x) + {p M + L_p +C \over p!} d_{p+1}(x - x_0) \\&+ a_k [f(x_{k+1}) + \langle \nabla f(x_{k+1}), x - x_{k+1} \rangle ]\\ \\\le & {} (A_k + a_k) f(x) + {p M + L_p +C \over p!} d_{p+1}(x - x_0), \end{aligned}$$

and this is $\mathcal{R}_{k+1}^2$. Let us show now that, for the appropriate choices of $a_k$, *C* and *M*, relation $\mathcal{R}_{k+1}^1$ is also valid.

Indeed, in view of $\mathcal{R}_k^1$ and Lemma 4 in [27], for any $x \in \mathbb {E}$, we have

3.5 $$\begin{aligned} \begin{array}{rcl} \psi _k(x) &{} \equiv &{} \ell _k(x) + {C \over p!} d_{p+1}(x-x_0) \; \ge \; \psi _k^* + {C \over (p+1)!} \cdot ({1 \over 2})^{p-1} \Vert x - v_k \Vert ^{p+1}\\ \\ &{} \ge &{} A_k f(x_k) + {C \over (p+1)!} \cdot ({1 \over 2})^{p-1} \Vert x - v_k \Vert ^{p+1}. \end{array} \end{aligned}$$

Denote $$\gamma _p = {C \over p!} \cdot ({1 \over 2})^{p-1}$$. Then,

$$\begin{aligned} \psi _{k+1}^*= & {} \min \limits _{x \in \mathbb {E}} \left\{ \psi _k(x) + a_k [f(x_{k+1}) + \langle \nabla f(x_{k+1}), x - x_{k+1} \rangle ] \right\} \\&\quad {\mathop {\ge }\limits ^{({\mathrm{3.5}})}} \min \limits _{x \in \mathbb {E}} \bigg \{ A_k f(x_k) + {\gamma _p \over p+1} \Vert x - v_k \Vert ^{p+1} + a_k [f(x_{k+1}) \\&\qquad +\langle \nabla f(x_{k+1}), x - x_{k+1} \rangle ] \bigg \}\\&\quad \ge \min \limits _{x \in \mathbb {E}} \bigg \{ (A_k + a_k)f(x_{k+1}) + A_k \langle \nabla f(x_{k+1}), x_k - x_{k+1} \rangle \\&\qquad + a_k \langle \nabla f(x_{k+1}), x - x_{k+1} \rangle + {\gamma _p \over p+1} \Vert x - v_k \Vert ^{p+1} \bigg \}\\&\quad {\mathop {=}\limits ^{({\mathrm{3.4}})}} \min \limits _{x \in \mathbb {E}} \bigg \{ A_{k+1} f(x_{k+1}) + \langle \nabla f(x_{k+1}), A_{k+1} y_k - a_k v_k - A_k x_{k+1} \rangle \\&\qquad + a_k \langle \nabla f(x_{k+1}), x - x_{k+1} \rangle + {\gamma _p \over p+1} \Vert x - v_k \Vert ^{p+1} \bigg \}\\&\quad = \min \limits _{x \in \mathbb {E}} \bigg \{ A_{k+1} f(x_{k+1}) + A_{k+1} \langle \nabla f(x_{k+1}), y_k - x_{k+1} \rangle \\&\qquad + a_k \langle \nabla f(x_{k+1}), x - v_k \rangle + {\gamma _p \over p+1} \Vert x - v_k \Vert ^{p+1} \bigg \}. \end{aligned}$$

Further, if we choose $M \ge L_p$, then by inequality (2.15) we have

$$\begin{aligned} \begin{array}{c} \langle \nabla f(x_{k+1}), y_k - x_{k+1} \rangle \; \ge \; {c(p) \over M} [M^2 - L_p^2]^{p-1 \over 2p} \, \Vert \nabla f(x_{k+1}) \Vert ^{p+1 \over p}_*. \end{array} \end{aligned}$$

Hence, our choice of parameters must ensure the following inequality:

$$\begin{aligned}&A_{k+1} {c(p) \over M} [M^2 - L_p^2]^{p-1 \over 2p} \, \Vert \nabla f(x_{k+1}) \Vert ^{p+1 \over p}_* + a_k \langle \nabla f(x_{k+1}), x - v_k \rangle \\&\quad + {\gamma _p \over p+1} \Vert x - v_k \Vert ^{p+1} \ge 0, \end{aligned}$$

for all $x \in \mathbb {E}$. Minimizing this expression in $x \in \mathbb {E}$, we come to the following condition:

$$\begin{aligned} \begin{array}{rcl} A_{k+1} {c(p) \over M} [M^2 - L_p^2]^{p-1 \over 2p}\ge & {} {p \over p+1} \left( {1 \over \gamma _p} \right) ^{1 \over p} a_k ^{p+1 \over p}. \end{array} \end{aligned}$$

Substituting in this inequality expressions for corresponding constants, we obtain

$$\begin{aligned} \begin{array}{rcl} A_{k+1} {p \over p+1} \left[ {p-1 \over p+1} \right] ^{1 - p \over 2p} \left[ (p-1)! \right] ^{1 \over p} {1 \over M} [M^2 - L_p^2]^{p-1 \over 2p}\ge & {} {p \over p+1} \left[ {p! \over C} 2^{p-1} \right] ^{1 \over p} a_k ^{p+1 \over p}. \end{array} \end{aligned}$$

After cancellations, we get

3.6 $$\begin{aligned} \begin{array}{rcl} A_{k+1} \sqrt{ 1 - {L_p^2 \over M^2}} \left( {C^2 \over M^2 - L_p^2}\right) ^{1 \over 2 p}\ge & {} 2 a_k^{p+1 \over p} \left( {p \over 2} \sqrt{p+1 \over p-1} \right) ^{1 \over p} \sqrt{p+1 \over p-1}. \end{array} \end{aligned}$$

For the sake of notation, let us choose

3.7 $$\begin{aligned} \begin{array}{rcl} C= & {} {p \over 2} \sqrt{{(p+1)\over (p-1)}(M^2 - L_p^2)}. \end{array} \end{aligned}$$

Then, inequality (3.6) becomes simpler:

3.8 $$\begin{aligned} \begin{array}{rcl} A_{k+1}\ge & {} 2 \sqrt{(p+1)M^2 \over (p-1)(M^2 - L_p^2)} \; a_k^{p+1 \over p} . \end{array} \end{aligned}$$

Let us choose some $\alpha > 0$ and define

3.9 $$\begin{aligned} \begin{array}{rcl} A_k &{} = &{} {1 \over \alpha } k^{p+1},\\ \\ a_k &{} = &{} A_{k+1} - A_k = {1 \over \alpha } ((k+1)^{p+1} - k^{p+1}). \end{array} \end{aligned}$$

Note that for any $k \ge 0$, using trivial inequality $(1-\tau )^p \ge 1 - p \tau$, $0 \le \tau \le 1$, we get

$$\begin{aligned} \begin{array}{rcl} {a_k \cdot A_{k+1}^{-{p \over p+1}}} &{} = &{} \alpha ^{-{1 \over p+1}} \cdot {(k+1)^{p+1} - k^{p+1} \over (k+1)^p} \; = \; \alpha ^{-{1 \over p+1}} \left( k + 1 - k \left( 1 - {1 \over k+1} \right) ^p \right) \\ \\ &{} \le &{} \alpha ^{-{1 \over p+1}} \left( 1 + {k p \over k+1} \right) \; \le \; \alpha ^{-{1 \over p+1}} (1 + p). \end{array} \end{aligned}$$

Thus, $A_{k+1} \ge \alpha ^{1 \over p} \left( {1 \over p+1} \right) ^{p+1 \over p} a_k^{p+1 \over p}$. Now, if we choose

3.10 $$\begin{aligned} \begin{array}{rcl} \alpha= & {} (p+1)^{p+1} \left[ {4(p+1)M^2 \over (p-1)(M^2 - L_p^2)} \right] ^{p \over 2}, \end{array} \end{aligned}$$

then $\alpha ^{-{1 \over p+1}} (1 + p) = \left[ { (p-1)(M^2 - L_p^2) \over 4(p+1)M^2} \right] ^{p \over 2(p+1)}$, and inequality (3.8) is satisfied for all $k \ge 0$.

Now we are ready to write down the accelerated tensor method. Define

3.11 $$\begin{aligned} \begin{array}{rcl} A_k= & {} \left[ {(p-1)(M^2 - L_p^2) \over 4 (p+1)M^2} \right] ^{p \over 2} \left( {k \over p+1} \right) ^{p+1}, \quad a_k \; = \; A_{k+1} - A_k, \quad k \ge 0. \end{array} \end{aligned}$$

3.12The above discussion proves the following theorem.

### Theorem 3

Let the sequence $\{x_k \}_{k=1}^{\infty }$ be generated by method (3.12) as applied to the problem (2.7). Then for any $k \ge 1$ we have:

3.13 $$\begin{aligned} \begin{array}{rcl} f(x_k) - f(x^*)\le & {} {p M + L_p + C \over (p+1)!} \left[ {4 (p+1)M^2 \over (p-1)(M^2 - L_p^2)} \right] ^{p \over 2} \left( {p+1 \over k} \right) ^{p+1} \Vert x_0 - x^* \Vert ^{p+1}. \end{array}\qquad \end{aligned}$$

### Proof

Indeed, we have shown that

$$\begin{aligned} \begin{array}{rcl} A_k f(x_k)&{\mathop {\le }\limits ^{\mathcal{R}_k^1}}&\psi _k^* \; {\mathop {\le }\limits ^{\mathcal{R}_k^2}} \; A_k f(x^*) + {p M + L_p + C \over (p+1)!} \Vert x_0 - x^* \Vert ^{p+1}. \end{array} \end{aligned}$$

Thus, (3.13) follows from (3.11). $\square$

Note that the point $v_k$ can be found in (3.12) by a closed-form expression. Consider

$$\begin{aligned} s_k = \nabla \ell _k(x). \end{aligned}$$

Since function $\ell _k(\cdot )$ is linear, this vector is the same for all $x \in \mathbb {E}$. Therefore, the point $v_k$ is a solution of the following minimization

$$\begin{aligned} \begin{array}{rcl} \min \limits _{x \in \mathbb {E}} \left\{ \langle s_k, x \rangle + {C \over (p+1)!} \Vert x - x_0 \Vert ^{p+1} \right\} . \end{array} \end{aligned}$$

The first-order optimality condition for this problem is as follows:

$$\begin{aligned} \begin{array}{rcl} s_k + {C \over p!} \Vert x - x_0 \Vert ^{p-1} B(x - x_0)= & {} 0. \end{array} \end{aligned}$$

Thus, we get the following closed-form expression for its solution:

$$\begin{aligned} \begin{array}{rcl} v_k= & {} x_0 - \left( {p! \over C \Vert s_k \Vert _*^{p-1}} \right) ^{1 \over p} \cdot B^{-1} s_k. \end{array} \end{aligned}$$

## Lower complexity bounds for tensor methods

For constructing functions, which are difficult for all tensor methods, it is convenient to assume that $\mathbb {E}= \mathbb {E}^* = {\mathbb {R}}^n$, and $B = I_n$, the identity $n \times n$-matrix. Thus, in this section we work with the standard Euclidean norm

$$\begin{aligned} \begin{array}{rcl} \Vert x \Vert= & {} \left[ \sum \limits _{i=1}^n (x^{(i)})^2 \right] ^{1/2}, \quad x \in {\mathbb {R}}^n. \end{array} \end{aligned}$$

For an integer parameter $p \ge 1$, define the following function:

$$\begin{aligned} \begin{array}{rcl} \eta _{p+1}(x)= & {} {1 \over p+1} \sum \limits _{i=1}^n | x^{(i)} |^{p+1}, \quad x \in {\mathbb {R}}^n. \end{array} \end{aligned}$$

Clearly, $\eta _{p+1} \in \mathcal{F}_p$. On the other hand, for any *x* and $h \in {\mathbb {R}}^n$, we have

$$\begin{aligned} \begin{array}{rcll} D^k \eta _{p+1}(x) [h]^k &{} = &{} {p! \over (p+1-k)!} \sum \limits _{i=1}^n | x^{(i)} |^{p+1-k} (h^{(i)})^k, &{}\text{ if } \text{ k } \text{ is } \text{ even, }\\ \\ D^k \eta _{p+1}(x) [h]^k &{} = &{} {p! \over (p+1-k)!} \sum \limits _{i=1}^n | x^{(i)} |^{p-k} x^{(i)} (h^{(i)})^k, &{}\text{ if } \text{ k } \text{ is } \text{ odd. } \end{array} \end{aligned}$$

Therefore, for all *x*, *y*, and *h* from ${\mathbb {R}}^n$, by Cauchy-Schwartz inequality we have

4.1 $$\begin{aligned} \begin{array}{rcl} | D^p \eta _{p+1}(x)[h]^p - D^p \eta _{p+1}(y)[h]^p| &{} \le &{} p! \Vert x - y \Vert \left[ \sum \limits _{i=1}^n (h^{(i)})^{2p} \right] ^{1/2}\\ \\ &{} \le &{} p! \; \Vert x - y \Vert \; \Vert h \Vert ^p. \end{array} \end{aligned}$$

Thus, $$L_p(\eta _{p+1}) = p!$$.

For integer parameter *k*, $2 \le k \le n$, let us define the following $k \times k$ upper triangular matrix with two nonzero diagonals:

$$\begin{aligned} \begin{array}{rcl} U_k &{} = &{} \left( \begin{array}{c@{\quad }r@{\quad }c@{\quad }c@{\quad }r} 1 &{} -1 &{} 0 &{} \dots &{} 0\\ 0 &{} 1 &{} -1&{} \dots &{} 0 \\ &{} &{} \dots &{} \dots &{} \\ 0 &{} 0 &{} \dots &{} 1 &{} -1 \\ 0 &{} 0 &{} \dots &{} 0 &{} 1 \end{array} \right) , \quad U_k^{-1} \; = \; \left( \begin{array}{c@{\quad }r@{\quad }c@{\quad }c@{\quad }r} 1 &{} 1 &{} 1 &{} \dots &{} 1\\ 0 &{} 1 &{} 1&{} \dots &{} 1 \\ &{} &{} \dots &{} \dots &{} \\ 0 &{} 0 &{} \dots &{} 1 &{} 1 \\ 0 &{} 0 &{} \dots &{} 0 &{} 1 \end{array} \right) \end{array} \end{aligned}$$

Now we can introduce $n \times n$ upper triangular matrix $A_k$ with the following structure:

$$\begin{aligned} \begin{array}{rcl} A_k &{} = &{} \left( \begin{array}{c@{\quad }c} U_k &{} 0 \\ 0 &{} I_{n-k} \end{array} \right) . \end{array} \end{aligned}$$

Note that

4.2 $$\begin{aligned} \Vert A_k \Vert ^2= & {} \max \limits _{x \in {\mathbb {R}}^n} \{ \Vert A x \Vert ^2: \; \Vert x \Vert \le 1 \} \nonumber \\= & {} \max \limits _{x \in {\mathbb {R}}^n} \left\{ \sum \limits _{i=1}^{k-1} (x^{(i)} - x^{(i+1)})^2 + \sum \limits _{i=k}^n (x^{(i)})^2 : \; \Vert x \Vert \le 1 \right\} \nonumber \\\le & {} \max \limits _{x \in {\mathbb {R}}^n} \left\{ \sum \limits _{i=1}^{k-1} \left[ 2(x^{(i)})^2 + 2 (x^{(i+1)})^2\right] + \sum \limits _{i=k}^n (x^{(i)})^2 : \; \Vert x \Vert \le 1 \right\} \nonumber \\\le & {} 4. \end{aligned}$$

Our parametric family of difficult functions is defined in the following way:

4.3 $$\begin{aligned} \begin{array}{rcl} f_k(x)= & {} \eta _{p+1}(A_k x) - \langle e_1, x \rangle , \quad 2 \le k \le p, \end{array} \end{aligned}$$

where $e_1 = (1, 0, \dots , 0)^T$. Let us compute its optimal solution from the first-order optimality condition

$$\begin{aligned} \begin{array}{rcl} A_k^T \nabla \eta _{p+1}(A_k x^*_k)= & {} e_1. \end{array} \end{aligned}$$

Thus, $A_k x^*_k = y^*_k$, where $y^*_k$ satisfies equation $$\nabla \eta _{p+1}(y^*_k) = A_k^{-T} e_1 = {\hat{e}}_k {\mathop {=}\limits ^{{\mathrm {def}}}}\left( \begin{array}{l} {\bar{e}}_k \\ 0_{n-k} \end{array} \right) $$, with ${\bar{e}}_k \in {\mathbb {R}}^k$ being the vector of all ones, and $0_{n-k}$ beng the origin in ${\mathbb {R}}^{n-k}$.

Thus, in coordinate form, vector $y^*_k$ can be found from the following equations:

$$\begin{aligned} \begin{array}{rcl} | (y^*_k)^{(i)} |^{p-2} (y^*_k)^{(i)} &{} = &{} 1, \quad i = 1, \dots , k,\\ \\ | (y^*_k)^{(i)} |^{p-2} (y^*_k)^{(i)} &{} = &{} 0, \quad i = k+1, \dots , n. \end{array} \end{aligned}$$

In other words, $y^*_k = {\hat{e}}_k$ and vector $x^*_k = A_k^{-1} {\hat{e}}_k$ has the following coordinates:

4.4 $$\begin{aligned} \begin{array}{rcl} (x^*_k)^{(i)}= & {} (k -i +1)_+, \quad i = 1, \dots , n, \end{array} \end{aligned}$$

where $$(\tau )_+ = \max \{ 0, \tau \}$$. Consequently,

4.5 $$\begin{aligned} \begin{array}{rcl} f^*_k &{} = &{} \eta _{p+1}({\hat{e}}_k) - \langle e_1 , x^*_k \rangle \; = \; {k \over p+1} - k \; = \; - {k p \over p+1},\\ \\ \Vert x^*_k \Vert ^2 &{} = &{} \sum \limits _{i=1}^k i^2 \; = \; {k(k+1)(2k+1) \over 6} \; \le \; {(k+1)^3 \over 3}. \end{array} \end{aligned}$$

Let us describe now the abilities of tensor methods of degree $p \ge 2$ in generating new test points. We assume that the response of oracle at point ${\bar{x}} \in {\mathbb {R}}^n$ consists in the following collection of multi-linear forms:

$$\begin{aligned} \begin{array}{c} f({\bar{x}}), \quad D^kf({\bar{x}})[h]^k, \quad k = 1, \dots , p. \end{array} \end{aligned}$$

Therefore, we assume that the method is able to compute stationary points of the following polynomial functions

4.6 $$\begin{aligned} \begin{array}{rcl} \phi _{a,\gamma ,m}(h)= & {} \sum \limits _{i=1}^p a^{(i)} D^i f({\bar{x}}) [h]^i + \gamma \Vert h \Vert ^m \end{array} \end{aligned}$$

with coefficients $a \in {\mathbb {R}}^p$, $\gamma > 0$ and $m > 1$. Denote by $\Gamma _{{\bar{x}}, f}(a,\gamma ,m)$ the set of all stationary points of this function. Then we can define the linear subspace

$$\begin{aligned} \begin{array}{rcl} \mathcal{S}_f({\bar{x}})= & {} \text{ Lin } \left( \Gamma _{{\bar{x}}, f}(a,\gamma ,m): \; a \in {\mathbb {R}}^p, \; \gamma> 0, \; m > 1 \right) . \end{array} \end{aligned}$$

Our assumption about the rules of tensor methods is as follows.

### Assumption 1

The method generates a sequence of test points $\{ x_k \}_{k \ge 0}$ satisfying recursive condition

4.7 $$\begin{aligned} x_{k+1} \in x_0 + \sum \limits _{i=0}^k \mathcal{S}_f(x_i), \quad k \ge 0. \end{aligned}$$

Note that for the absolute majority of the first-order, second-order, and tensor methods this assumption is satisfied.

Let us look at the consequences Assumption 1 in the case of minimization of function $f_k(\cdot )$. Denote

$$\begin{aligned} \begin{array}{rcl} {\mathbb {R}}_k^n= & {} \{ x \in {\mathbb {R}}^n: \; x^{(i)} = 0, \; i = k+1, \dots , n \}, \quad 1 \le k \le n-1. \end{array} \end{aligned}$$

### Lemma 2

Any tensor method satisfying Assumption 1 and minimizing function $f_t(\cdot )$ starting from the point $x_0 = 0$, generates test points $\{ x_k \}_{k \ge 0}$ satisfying condition

4.8 $$\begin{aligned} \begin{array}{rcl} x_{k+1}\in & {} \sum \limits _{i=0}^{k} \mathcal{S}_{f_t}(x_i) \; \subseteq \; {\mathbb {R}}^n_{k+1}, \quad 0 \le k \le t-1. \end{array} \end{aligned}$$

### Proof

Let us prove first that inclusion $x \in {\mathbb {R}}^n_k$ with $k \ge 1$ implies $\mathcal{S}_{f_t}(x) \subseteq {\mathbb {R}}^n_{k+1}$. Indeed, since matrix $A_t$ is upper triangular, this inclusion ensures that $y {\mathop {=}\limits ^{{\mathrm {def}}}}A_t x \in {\mathbb {R}}^n_k$. Therefore, all derivatives of function $f_t(\cdot )$ along direction $h \in {\mathbb {R}}^n$ have the following form:

$$\begin{aligned} \begin{array}{rcl} D f_t(x)[h] &{} = &{} D\eta _{p+1}(y) [A_t h] - h^{(1)} \; = \; \sum \limits _{i=1}^k d_{i,1} \langle e_i, A_t h \rangle - h^{(1)},\\ \\ D^i f_t(x)[h]^k &{} = &{} D^k \eta _{p+1}(y) [A_t h]^k \; = \; \sum \limits _{i=1}^k d_{i,k} \langle e_i, A_t h \rangle ^k, \quad 2 \le k \le p, \end{array} \end{aligned}$$

with certain coefficients $d_{i,k}$, $i = 1, \dots , n$, $k = 1, \dots , p$. This means that the gradients of these derivatives in *h* are as follows

$$\begin{aligned} \begin{array}{rcl} \nabla D f_t(x)[h] &{} = &{} \sum \limits _{i=1}^k d_{i,1} A_t^Te_i - e_1,\\ \\ \nabla D^k f_t(x)[h]^k &{} = &{} \sum \limits _{i=1}^k k d_{i,k} \langle e_i, A_t h \rangle ^{k-1} A_t^T e_i, \quad 2 \le k \le p, \end{array} \end{aligned}$$

Thus $\nabla D^k f_t(x)[h]^k \in {\mathbb {R}}^n_{k+1}$ for all *k*, $1 \le k \le p$. Hence, since the regularization term in definition (4.6) is formed by the standard Euclidean norm, all stationary points of this function belong to ${\mathbb {R}}^n_{k+1}$.

Now we can prove statement of the lemma by induction. For $k=0$, we have $x_0 = 0$, and therefore

$$\begin{aligned} \begin{array}{rcl} \nabla f_t(x_0)= & {} - e_1, \quad D^if(x_0) [h]^i \; = \; 0, \quad i = 1, \dots , p, \end{array} \end{aligned}$$

for all $h \in {\mathbb {R}}^n$. Consequently, stationary points of all possible functions $\phi _{a,\gamma ,m}(\cdot )$ belong to ${\mathbb {R}}^n_1$ implying $\mathcal{S}_{f_t}(x_0) = {\mathbb {R}}^n_1$. Thus, $x_1$ belongs to ${\mathbb {R}}^n_1$ by Assumption 1.

Assume now that all $x_i \in {\mathbb {R}}^n_k$, $i = 1, \dots , k$, for some $k \ge 1$. Then, as we have already seen, $\mathcal{S}_{f_t}(x_i) \subseteq {\mathbb {R}}^n_{k+1}$. Hence, the inclusion (4.8) follows from Assumption 1. $\square$

Now we can prove the main statement of this section.

### Theorem 4

Let some tensor method $\mathcal{M}$ of degree *p* satisfies Assumption 1. Assume that this method ensures for any function $f \in \mathcal{F}_p$ with $L_p((f) < + \infty$ the following rate of convergence:

4.9 $$\begin{aligned} \begin{array}{rcl} \min \limits _{0 \le k \le t} f(x_k) - f_*\le & {} {L_p \Vert x_0 - x^* \Vert ^{p+1} \over (p+1)!\; \kappa (t)}, \quad t \ge 1, \end{array} \end{aligned}$$

where $\{ x_k \}_{k \ge 0}$ is the sequence of test points, generated by method $\mathcal{M}$ and $x^*$ is the solution of the problem (2.7). Then for all $t \ge 1$ such that $2t +1 \le n$ we have

4.10 $$\begin{aligned} \begin{array}{rcl} \kappa (t)\le & {} {1 \over 3 p} 2^{p+1} \, (2t+2)^{3p+1 \over 2}. \end{array} \end{aligned}$$

### Proof

Let us use method $\mathcal{M}$ for minimizing function $f(x) = f_{2t+1}(x)$. In view of Lemma 2, we have $x_i \in {\mathbb {R}}^n_t$ for all *i*, $0 \le i \le t$. However,

$$\begin{aligned} \begin{array}{rcl} f_{2t+1}(x)\equiv & {} f_t(x), \quad \forall x \in {\mathbb {R}}^n_t. \end{array} \end{aligned}$$

At the same time, for all $x, y, h \in {\mathbb {R}}^n$ we have

$$\begin{aligned}&|D^pf_{2t+1}(x)[h]^p - D^p f_{2t+1}(y)[h]^p| \\&\quad = |D^p\eta _{p+1}(x)[A_{2t+1}h]^p - D^p \eta _{p+1}(y)[A_{2t+1} h]^p| \\&\quad {\mathop {\le }\limits ^{({\mathrm{4.1}})}} p! \Vert x - y \Vert \Vert A_{2t+1} h \Vert ^p \\&\quad {\mathop {\le }\limits ^{({\mathrm{4.2}})}} 2^p p! \Vert x - y \Vert . \end{aligned}$$

Therefore, $L_p(f_{2t+1}) \le 2^p p!$, and we have

$$\begin{aligned} \begin{array}{rcl} (p+1)! \; \kappa (t) &{} {\mathop {\le }\limits ^{({\mathrm{4.9}})}} &{} {L_p(f_{2t+1}) \Vert x_0 - x^*_{2t+1} \Vert ^{p+1} \over \min \limits _{0 \le k \le t} f(x_k) - f^*_{2t+1} } \; \le \; {2^p p! (2t+2)^{{3 \over 2}(p+1)} \over 3(f^*_t - f^*_{2t+1})} \\ \\ &{} = &{} {2^p p! (2t+2)^{{3 \over 2}(p+1)} \over 3 (t+1) } \cdot {p+1 \over p}. \end{array} \end{aligned}$$

$$\square $$

## Third-order methods: implementation details

Tensor optimization methods, presented in Sects. 2 and 3, are based on the solution of the auxiliary optimization problem (2.6). In the existing literature on the tensor methods [6, 7, 20, 32], it was solved by the standard local technique of Nonconvex Optimization. However, now we know that by Theorem 1, this problem is convex. Hence, it is solvable by the standard and very efficient methods of Convex Optimization.

Since we need to solve this problem at each iteration of the methods, its complexity significantly affects the total computational time. Since the objective function in the problem (2.6) is a *convex multivariate polynomial*, we there could exist some special efficient algorithms for finding its solution. Unfortunately, at this moment the authors failed to find such methods in the literature. Therefore, we present in this section a special approach for solving the problem (2.6) with the third degree Taylor approximation, which is based on the recently developed optimization framework of *relatively smooth functions* (see [4, 21]).

Let us fix an arbitrary $x \in \mathbb {E}$. Consider the following multivariate polynomial of degree three:^2^

$$\begin{aligned} \begin{array}{rcl} \Phi _{x}(h)= & {} \langle \nabla f(x), h \rangle + {1 \over 2}\langle \nabla ^2 f(x)h, h \rangle + {1 \over 6} D^3 f(x) [h]^3. \end{array} \end{aligned}$$

Since in this section we work only with third-order approximations, we drop the corresponding index.

Recall that $D^3f(x)[h_1,h_2,h_3]$ is a symmetric trilinear form. Hence, $D^3 f(x) [h_1,h_2] \in \mathbb {E}^*$ is a symmetric bilinear vector function, and $D^3f(x)[h]$ is a linear function of $h \in \mathbb {E}$, whose values are self-adjoint linear operators from $\mathbb {E}$ to $\mathbb {E}^*$ (as Hessians).

Denote $p(h) = {1 \over 6} D^3 f(x) [h]^3$. Then we can define its gradient and Hessian as follows:

5.1 $$\begin{aligned} \begin{array}{rcl} \nabla p(h)= & {} {1 \over 2}D^3f(x)[h,h], \quad \nabla ^2 p(h) \; = \; D^3 f(x)[h]. \end{array} \end{aligned}$$

In this section, our main class of functions is $\mathcal{F}_3$, composed by convex functions, which are three times continuously differentiable, and for which the constant $L_3$ is finite. As we have shown in Theorem 1, our assumptions imply interesting relations between derivatives. Let us derive a consequence of the matrix inequality (2.3).

### Lemma 3

For any $h \in \mathbb {E}$ and $\tau > 0$, we have

5.2 $$\begin{aligned} \begin{array}{rcl} - {1 \over \tau }\nabla ^2 f(x) - { \tau \over 2 } L_3 \Vert h \Vert ^2 B \; \preceq \; D^3f(x)[h]\preceq & {} {1 \over \tau }\nabla ^2 f(x) + { \tau \over 2 } L_3 \Vert h \Vert ^2 B. \end{array} \end{aligned}$$

Consequently, for any $h, u \in \mathbb {E}$, we get

5.3 $$\begin{aligned} \begin{array}{rcl} D^3 f(x)[h,u,u]\le & {} \sqrt{2L_3} \langle \nabla ^2 f(x) u,u \rangle ^{1/2} \Vert u \Vert \Vert h \Vert . \end{array} \end{aligned}$$

### Proof

Let us fix arbitrary directions $u, h \in \mathbb {E}$. Then, in view of relation (2.3) we have

$$\begin{aligned} \begin{array}{rcl} 0 &{} \le &{} \langle \nabla ^2 f(x + h) u, u \rangle \; \le \; \langle \nabla ^2 \Phi _x(h) u, u \rangle + {1 \over 2}L_3 \Vert h \Vert ^2 \Vert u \Vert ^2\\ \\ &{} = &{} \langle (\nabla ^2 f(x) + D^3f(x)[h]) u, u \rangle + {1 \over 2}L_3 \Vert h \Vert ^2 \Vert u \Vert ^2 \end{array} \end{aligned}$$

Thus, replacing *h* by $\tau h$ with $\tau > 0$ and dividing the resulting inequality by $\tau$, we get

$$\begin{aligned} \begin{array}{rcl} - \langle D^3f(x)[h]u,u \rangle\le & {} {1 \over \tau } \langle \nabla ^2 f(x) u,u \rangle + { \tau \over 2 } L_3 \Vert h \Vert ^2 \Vert u \Vert ^2. \end{array} \end{aligned}$$

And this is equivalent to the left-hand side of matrix inequality (5.2). Its right-hand side can be obtained by replacing *h* by $-h$, which gives

$$\begin{aligned} \begin{array}{rcl} \langle D^3f(x)[h]u, u \rangle\le & {} {1 \over \tau }\langle \nabla ^2 f(x)u, u \rangle + { \tau \over 2 } L_3 \Vert h \Vert ^2 \Vert u \Vert ^2. \end{array} \end{aligned}$$

Minimizing the right-hand side of this inequality in $\tau$, we get (5.3). $\square$

Let us look now at our auxiliary minimization problem:

5.4 $$\begin{aligned} \begin{array}{rcl} \Omega _{x,M}(h)&{\mathop {=}\limits ^{{\mathrm {def}}}}&\Phi _x(h) + {M \over 2} d_4(h) \; \rightarrow \; \min \limits _{h \in \mathbb {E}}, \end{array} \end{aligned}$$

where $d_4(h) = {1 \over 4} \Vert h \Vert ^4$. In view of Theorem 1, function $\Omega _{x,M}(\cdot )$ is convex for

5.5 $$\begin{aligned} \begin{array}{rcl} M= & {} \tau ^2 L_3 \end{array} \end{aligned}$$

with $\tau \ge 1$. For any $h \in \mathbb {E}$, we have

$$\begin{aligned} \begin{array}{rcl} \nabla ^2 \Omega _{x,M}(h) &{} = &{} \nabla ^2 f(x) + D^3f(x)[h] + {M \over 2} \nabla ^2 d_4(h) \\ \\ &{} {\mathop {\succeq }\limits ^{({\mathrm{5.2}})}} &{} (1 - {1 \over \tau }) \nabla ^2 f(x) + {M \over 2} \nabla ^2 d_4(h) - {\tau \over 2}L_3 \Vert h \Vert ^2 B\\ \\ &{} {\mathop {\succeq }\limits ^{({\mathrm{2.2}})}} &{} (1 - {1 \over \tau }) \nabla ^2 f(x) + {M -\tau L_3 \over 2} \nabla ^2 d_4(h). \end{array} \end{aligned}$$

Let $\rho _x(h) = {1 \over 2}(1 - {1 \over \tau }) \langle \nabla ^2 f(x) h,h \rangle + {M -\tau L_3 \over 2} d_4(h)$. Then, we have proved that

5.6 $$\begin{aligned} \begin{array}{rcl} \nabla ^2 \Omega _{x,M}(h)\succeq & {} \nabla ^2 \rho _x(h), \quad h \in \mathbb {E}. \end{array} \end{aligned}$$

On the other hand,

$$\begin{aligned} \begin{array}{rcl} \nabla ^2 \Omega _{x,M}(h) &{} = &{} \nabla ^2 f(x) + D^3f(x)[h] + {M \over 2} \nabla ^2 d_4(h) \\ \\ &{} {\mathop {\preceq }\limits ^{({\mathrm{5.2}})}} &{} (1 + {1 \over \tau }) \nabla ^2 f(x) + {M \over 2} \nabla ^2 d_4(h) + {\tau \over 2}L_3 \Vert h \Vert ^2 B\\ \\ &{} {\mathop {\preceq }\limits ^{({\mathrm{2.2}})}} &{} (1 + {1 \over \tau }) \nabla ^2 f(x) + {M + \tau L_3 \over 2} \nabla ^2 d_4(h)\\ \\ &{} {\mathop {=}\limits ^{({\mathrm{5.5}})}} &{} \left( {1 + \tau \over \tau -1}\right) \left( (1 - {1 \over \tau }) \nabla ^2 f(x) + {\tau (\tau -1) L_3 \over 2} \nabla ^2 d_4(h) \right) \\ \\ &{} = &{} {\tau +1 \over \tau - 1} \nabla ^2 \rho _x(h). \end{array} \end{aligned}$$

Thus, we have proved the following lemma.

### Lemma 4

Let $M = \tau ^2L_3$ with $\tau > 1$. Then function $\Omega _{x,M}(\cdot )$ satisfies the strong relative smoothness condition

5.7 $$\begin{aligned} \begin{array}{rcl} \nabla ^2 \rho _x(h)\preceq & {} \nabla ^2 \Omega _{x,M}(h) \; \preceq \; \kappa (\tau ) \nabla ^2 \rho _x(h), \quad h \in \mathbb {E}, \end{array} \end{aligned}$$

with respect to function $\rho _x(\cdot )$, where $\kappa (\tau )={\tau +1 \over \tau -1}$.

As it is shown in [21], condition (5.7) allows to solve problem (5.4) very efficiently by a kind of primal gradient method. In accordance to this approach, we need to define the *Bregman distance* of function $\rho _x(\cdot )$:

$$\begin{aligned} \begin{array}{rcl} \beta _{\rho _x}(u,v) &{} = &{} \rho _x(v) - \rho _x(u) - \langle \nabla \rho _x(u), v - u \rangle \\ \\ &{} = &{} {1 \over 2}(1 - {1 \over \tau }) \langle \nabla ^2 f(x)(v-u),v-u\rangle + {\tau (\tau -1) \over 2} L_3 \beta _{d_4}(u,v), \end{array} \end{aligned}$$

and iterate the process

$$\begin{aligned} \begin{array}{rcl} h_{k+1}= & {} \arg \min \limits _{h \in \mathbb {E}} \left\{ \langle \nabla \Omega _{x,M}(h_k), h - h_k \rangle + \kappa (\tau ) \beta _{\rho _x}(h_k,h) \right) . \end{array} \end{aligned}$$

In our case, this method has the following form:

5.8 $$\begin{aligned} h_0= & {} 0, \nonumber \\ h_{k+1}= & {} \arg \min \limits _{h \in \mathbb {E}} \Big \{ \langle \nabla \Omega _{x,M}(h_k) , h - h_k \rangle \nonumber \\&+ {\tau +1 \over 2} \left[ {1 \over \tau } \langle \nabla ^2 f(x) (h - h_k), h- h_k \rangle + \tau L_3 \beta _{d_4}(h_k,h) \right] \Big \}, \quad k \ge 0.\qquad \quad \end{aligned}$$

In accordance to Theorem 3.1 in [21], the rate of convergence of this method is as follows:

5.9 $$\begin{aligned} \begin{array}{rcl} \Omega _{x,M}(h_k) - \Omega _{x,M}(h_*)\le & {} {\beta _{\rho _x}(h_0,h_*) \over \left( {\kappa (\tau ) \over \kappa (\tau )-1} \right) ^k-1}\; = \; {{\tau - 1 \over 2} [{1 \over \tau } \langle \nabla ^2 f(x) h_*, h_* \rangle + {\tau \over 4} L_3 \Vert h_* \Vert ^4] \over \left( {\tau + 1 \over 2} \right) ^k - 1}, \end{array} \end{aligned}$$

where $h_*$ is the unique optimal solution to problem (5.4).

As we can see, the algorithm (5.8) is very fast. Its linear rate of convergence depends only on absolute constant $\tau > 1$, which can be chosen reasonably close to one for allowing faster convergence of the main tensor methods (2.16) and (3.12). Let us discuss two potentially expensive operations in the implementation of method (5.8).

1. **Computation of the gradient** $\nabla \Omega _{x,M}(h)$. Note that
  $$\begin{aligned} \begin{array}{rcl} \nabla \Omega _{x,M}(h)= & {} \nabla f(x) + \nabla ^2 f(x) h + {1 \over 2}D^3f(x) [h]^2. \end{array} \end{aligned}$$
  In this formula, only the computation of the third derivative may be dangerous. However, this difficulty can be resolved using the technique of automatic differentiation (see, for example, [19]). Indeed, assume we have a sequence of operations for computing the function value *f*(*x*) with computational complexity *T*. Let us fix a direction $h \in \mathbb {E}$. Then by forward differentiation, we can generate automatically a sequence of operations for computing the value
  $$\begin{aligned} \begin{array}{rcl} g_h(x)= & {} \langle \nabla ^2 f(x) h, h \rangle \end{array} \end{aligned}$$
  with computational complexity *O*(*T*). Now, by backward differentiation in *x*, we can compute the gradient of this function:
  $$\begin{aligned} \begin{array}{rcl} \nabla g(x)= & {} D^3f(x)[h,h] \end{array} \end{aligned}$$
  with computational complexity *O*(*T*). Thus, the oracle complexity of method (5.8) is proportional to the complexity of computing the function value *f*(*x*). Another example of simple computation of the third derivative is provided by a separable objective function. Assume that $\mathbb {E}= {\mathbb {R}}^n$ and
  $$\begin{aligned} \begin{array}{rcl} f(x)= & {} \sum \limits _{i=1}^N f_i(b_i - \langle a_i, x \rangle ), \end{array} \end{aligned}$$
  where $a_i \in {\mathbb {R}}^n$ and univariate functions $f_i(\cdot )$ are three times continuously differentiable, $i = 1, \dots , N$. Then vector $D^3 f[h]^2$ has the following representation:
  $$\begin{aligned} \begin{array}{rcl} D^3 f(x)[h]^2= & {} - \sum \limits _{i=1}^N a_i \, f'''_i(b_i - \langle a_i, x \rangle ) \langle a_i, h \rangle ^2. \end{array} \end{aligned}$$
  Thus, for solving the problem (5.4), we need to compute in advance all values
  $$\begin{aligned} \begin{array}{c} f'''_i(b_i - \langle a_i, x \rangle ), \quad i = 1, \dots , N \end{array} \end{aligned}$$
  (this needs *O*(*nN*) operations). After that, each computation of vector $D^3 f(x)[h]^2 \in {\mathbb {R}}^n$ also needs *O*(*nN*) operations. This computation will be cheaper for the sparse data.
2. **Solution of the auxiliary problem** At all iterations of method (5.8), we need to solve an auxiliary problem in the following form:
  5.10 $$\begin{aligned} \begin{array}{rcl} \min \limits _{h \in \mathbb {E}} \left\{ \langle c, h \rangle + {1 \over 2}\langle A h, h \rangle + {\gamma \over 4} \Vert h \Vert ^4 \right\} , \end{array} \end{aligned}$$
  where $A \succeq 0$ and $\gamma > 0$. Note that at all these iterations only the vector *c* and coefficients $\gamma$ are changing, and matrix $A = \nabla ^2 f(x)$ remains the same. Therefore, before the algorithm (5.8) starts working, it is reasonable to transform this matrix in a tri-diagonal form:
  $$\begin{aligned} \begin{array}{rcl} A= & {} U T U^T, \end{array} \end{aligned}$$
  where $U \in {\mathbb {R}}^{n \times n}$ is an orthogonal matrix: $UU^T = I$, and $T \in {\mathbb {R}}^{n \times n}$ is tri-diagonal.

Denoting now ${\tilde{c}} = U^T c$, we have:

5.11 $$\begin{aligned}&\min \limits _{h \in \mathbb {E}} \left\{ \langle c, h \rangle + {1 \over 2}\langle U T U^T h, h \rangle + {\gamma \over 4} \Vert h \Vert ^4 \right\} \nonumber \\&\quad = \min \limits _{h \in \mathbb {E}} \max \limits _{\tau> 0} \left\{ \langle {\tilde{c}}, U^T h \rangle + {1 \over 2}\langle T U^Th, U^Th \rangle + {\gamma \over 2} \tau \Vert U^Th \Vert ^2 - {1 \over 2}\tau ^2 \right\} \nonumber \\&\quad = \max \limits _{\tau> 0} \min \limits _{h \in \mathbb {E}} \left\{ \langle {\tilde{c}}, U^T h \rangle + {1 \over 2}\langle T U^Th, U^Th \rangle + {\gamma \over 2} \tau \Vert U^Th \Vert ^2 - {1 \over 2}\tau ^2 \right\} \nonumber \\&\quad = - \min \limits _{\tau > 0} \left\{ {1 \over 2}\tau ^2 + {1 \over 2}\langle (\gamma \tau I + T)^{-1} {\tilde{c}}, {\tilde{c}} \rangle \right\} . \end{aligned}$$

Thus, the solution of the primal problem can be retrieved from a solution to the univariate dual problem. The complexity of computing its function value and derivative is linear in *n*. Moreover, since its objective function is strongly convex and infinitely times differentiable, all reasonable one-dimensional methods have global linear rate of convergence and the quadratic convergence in the end.

Let us estimate the total computational complexity of the method (5.8), assuming that the computational time of the value of the objective function is $T_f$. Assume also that its gradient, the product of its Hessian by a vector, and the value of its third derivative on two identical vectors can be computed using the fast backward differentiation (then the complexity of all these operations is $O(T_f)$). Then, the most expensive operations in this method are as follows.

- Computation of the Hessian $\nabla ^2 f(x)$ and its tri-diagonal factorization: $O(nT_f + n^3)$ operations.
- We need $O(\ln {1 \over \epsilon })$ iterations of method (5.8), in order to get $\epsilon$-solution of the auxiliary problem. At each iteration of this method we need:
  - Compute the gradient $\nabla \Omega _{x,M}(h_k)$: $O(T_f)$ operations.
  - Compute the vector ${\tilde{c}}$ for the univariate problem in (5.11): $O(n^2)$ operations.
  - Solve the dual problem in (5.11) up to accuracy $\delta$: $O(n \ln {1 \over \delta })$ operations.
  - Compute an approximate solution $h = - U(\gamma \tau I +T)^{-1} {\tilde{c}}$ of the problem (5.10), using an approximate solution $\tau$ of the dual problem: $O(n^2)$ operations.

Thus, we come to the following estimate:

$$\begin{aligned} \begin{array}{c} O\left( nT_f + n^3 + \left[ T_f + n^2 + n \ln {1 \over \delta } \right] \ln {1 \over \epsilon } \right) . \end{array} \end{aligned}$$

This is the same order of complexity as that of one iteration in Trust Region Methods [15] and usual Cubic Regularization [27, 31]. However, we can expect that the third-order methods converge much faster.

For the readers, which are not interested in all these computational details, we just mention that the Galahad Optimization Library [16] has special subroutines for solving the auxiliary problems in the form (5.10).

## Discussion

In this paper, we did an important step towards practical implementation of tensor methods in unconstrained convex optimization. We have shown that the auxiliary optimization problems in these scheme can be reduced to minimization of a convex multivariate polynomial. In the important case of third-order tensor, we have proved that this problem can be efficiently solved by a special optimization scheme derived from the relative smoothness condition.

Our results highlight several interesting questions. One of the direct consequences of our approach is a systematic way of generating convex multivariate polynomials. Is it possible to minimize them by some tools of Algebraic Geometry (see [23] for the related technique like sums of squares, etc.), or we need to treat them using an appropriate technique from Convex Optimization? The results of Sect. 5 demonstrate a probably unbeatable superiority of optimization technique for the third-order polynomials. But what happens with polynomials of higher degree?

One of the difficult unsolved problems in our approach is related to dynamic adjustment of the Lipschitz constant for the highest derivative. This dynamic estimate should not be much bigger than the actual Lipschitz constant. On the other hand, it must ensure convexity of the auxiliary problem solved at each iteration of the tensor methods. This question is clearly crucial for the practical efficiency of the high-order schemes.

Simple comparison of the complexity bounds in Sects. 3 and 4 shows that we failed to develop an optimal tensor scheme. The missing factor in the complexity estimates is of the order of $O\left( \left( {1 \over \epsilon }\right) ^{{1 \over p+1} - {2 \over 3p + 1}}\right) = O\left( \left( {1 \over \epsilon }\right) ^{p-1 \over (p+1)(3p+1)}\right)$. For $p = 3$, this factor is of the order of $O\left( \left( {1 \over \epsilon }\right) ^{{1 \over 20}}\right)$. This means that from the viewpoint of practical efficiency, the cost of one iteration of the hypothetical optimal scheme must be of the same order as that of the accelerated tensor method (3.12). Any additional logarithmic factors in the complexity bound of this “optimal” method will definitely kill its tiny superiority in the convergence rate.

In the last years, we have seen an increasing interest to universal methods, which can adjust to the best possible Hölder condition instead of the Lipschitz one during the running optimization process (see [14, 17, 29]). Of course, it is very interesting to extend this philosophy onto the tensor minimization schemes. Another important extension could be the treatments of the constraints, either in functional form, or using the framework of composite minimization [28]. The main difficulty here is related to the complexity of the auxiliary optimization problems.

One of the main restrictions for practical implementation of our results is the necessity to know the Lipschitz constant of the corresponding derivative. If our estimate is too small, then the auxiliary problem (2.6) may loose convexity. Consequently, we will loose the fast convergence in the auxiliary process (5.8). However, this observation gives us a clue how to tune this constant: if we see that this process is too slow, this means that our estimate is too small. But of course it is very interesting to find a recipe with better theoretical justification.

## Footnotes

1 In our notation, the approximation function in [3] was chosen as $\Phi _{x,p}(y) + {M \over p!} d_{p+1}(y-x)$. Thus, we cannot guarantee that this polynomial is convex.

2 We drop the index in this notation since from now on we always have $p = 3$.
